# Salvage robotic-assisted radical prostatectomy in a patient with recurrence after two sessions of heavy ion radiotherapy

**DOI:** 10.1093/jscr/rjae512

**Published:** 2024-08-19

**Authors:** Ryo Yabusaki, Ayako Arai, Yu Odagaki, Jun Nakashima, Ken Nakamura, Hitoshi Ishikawa, Noriyuki Okonogi, Shuri Aoki, Hiroshi Tsuji, Choichiro Ozu

**Affiliations:** Department of Urology, Sanno Hospital, 8-10-16 Akasaka, Minato-ku, Tokyo 174-0052, Japan; Department of Urology, Sanno Hospital, 8-10-16 Akasaka, Minato-ku, Tokyo 174-0052, Japan; Department of Urology, Itabashi Chuo Medical Center, 2-12-7 Azusawa, Itabashi-ku, Tokyo 174-0051, Japan; Department of Urology, Sanno Hospital, 8-10-16 Akasaka, Minato-ku, Tokyo 174-0052, Japan; Department of Urology, National Hospital Organization Tokyo Medical Center, 2-5-1 Higashigaoka, Meguro-ku, Tokyo 152-8902, Japan; Department of Prostate, National Institutes for Quantum Science and Technology Hospital, 4-9-1 Anagawa, Inage-ku, Chiba 263-0024, Japan; Department of Radiation Oncology, Juntendo University Graduate School of Medicine, 2-1-1 Hongo, Bunkyo-ku 113-8421, Tokyo, Japan; Department of Prostate, National Institutes for Quantum Science and Technology Hospital, 4-9-1 Anagawa, Inage-ku, Chiba 263-0024, Japan; Department of Prostate, National Institutes for Quantum Science and Technology Hospital, 4-9-1 Anagawa, Inage-ku, Chiba 263-0024, Japan; Department of Urology, Sanno Hospital, 8-10-16 Akasaka, Minato-ku, Tokyo 174-0052, Japan

**Keywords:** salvage radical prostatectomy, salvage robotic-assisted radical prostatectomy, heavy-ion radiotherapy

## Abstract

Salvage radical prostatectomy is a postradiation treatment for patients with localized prostate cancer. In 2016, Ozu *et al.* (Ozu C, Aoki K, Nakamura K, Yagi Y, Muro Y, Nishiyama T, *et al.* The initial case report: salvage robotic assisted radical prostatectomy after heavy ion radiotherapy. *Urol Case Rep* 2016;**7**:45-7) first reported salvage robotic-assisted radical prostatectomy (sRARP) after heavy-ion radiotherapy (HIRT). Thereafter, sRARP has been performed in >100 cases. However, it is currently avoided owing to some difficulties. Herein, we report about sRARP in a 67-year-old man who received two sessions of HIRT despite some expected challenges. He was initially treated with HIRT for prostate cancer in 2009 and received the second HIRT as salvage treatment for local recurrence in 2016. In 2019, he had biochemical recurrence and underwent sRARP. There were no significant peri- or postoperative complications. Subsequently, 12 months after sRARP, hormonal therapy was introduced after the diagnosis of biochemical recurrence. The patient’s prostate-specific antigen level is currently undetectable.

## Introduction

Heavy-ion radiotherapy (HIRT) is a treatment option for localized prostate cancer and sRARP is a postradiation, including HIRT, treatment [1]. The prostate-specific antigen (PSA) recurrence rate after HIRT in patients with prostate cancer is extremely low. However, the number of cases of local recurrence after HIRT is low, and no clear treatment guidelines for local recurrence are available. In recent reports, salvage robotic-assisted radical prostatectomy (sRARP) for radiation therapy (including intensity modulated radiation therapy and brachytherapy) or hormone therapy for localized prostate cancer is extremely effective. In general, sRARP is avoided because it is associated with a poor functional prognosis and high complication rate. Herein, we report the first case of sRARP after two sessions of HIRT.

## Case report

**Figure 1:**
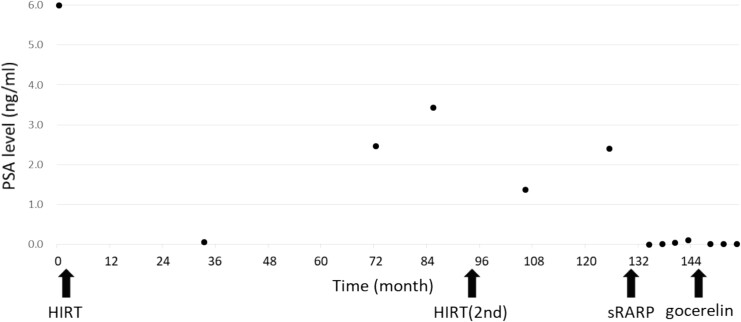
The patient’s serum prostate-specific antigen (PSA) level before heavy-ion therapy (HIRT). His PSA level before the HIRT was 5.99 ng/ml. Biochemical progression was observed after two sessions of the HIRT. His PSA level decreased below detection sensitivity after salvage robotic-assisted radical prostatectomy.

A 67-year-old man was referred to our hospital for prostate cancer treatment. His initial serum PSA level was 5.99 ng/ml (Fig. 1), and the patient underwent transrectal prostate biopsy in 2009. The pathological finding in 3 of 10 sites was adenocarcinoma (Gleason score 3 + 4). Metastatic lesions were not observed on computed tomography (CT) and bone scans. The patient was diagnosed with T1cN0M0 prostate cancer. He selected combination therapy with androgen deprivation therapy (ADT) and HIRT.

He was treated with bicalutamide as ADT. Subsequently, he received HIRT (57.6 Gy/12 fractions) in 2009. ADT was administered for 6 months. His PSA levels decreased to 0.06 ng/ml and remained undetectable. However, the PSA levels gradually increased since 2012.

**Figure 2:**
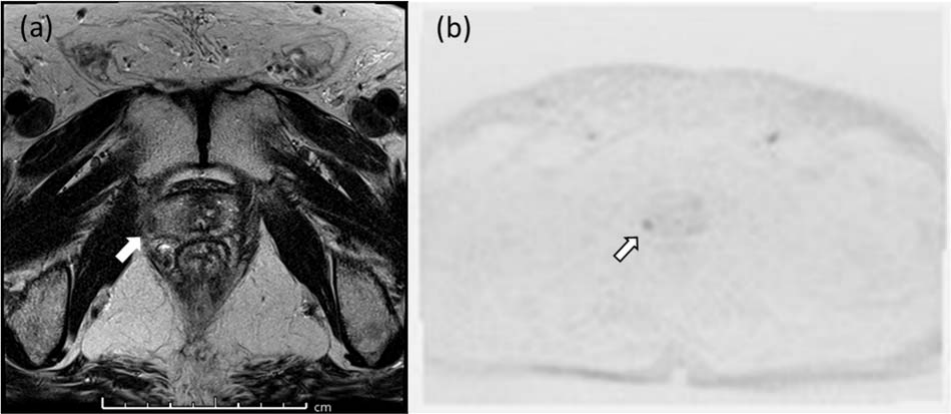
Pelvic magnetic resonance imaging revealed a lesion in the right peripheral zone of the prostate (a, b).

In 2015, his PSA level increased to 2.461 ng/ml, and he was diagnosed with biochemical recurrence. Magnetic resonance imaging (MRI) revealed a lesion in the right peripheral zone of the prostate (Fig. 2). In 2016, his PSA level increased to 3.427 ng/ml. As there was no metastasis, salvage HIRT was indicated, and he again received HIRT (51.6 Gy/12 fractions).

**Figure 3:**
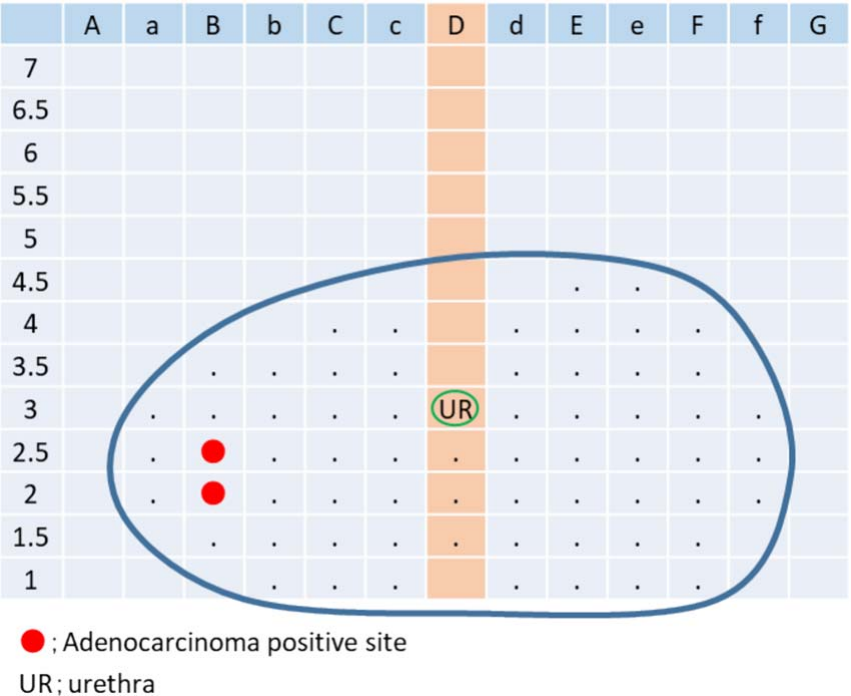
Positive site of template needle biopsies.

Thereafter, the treatment went well. However, in 2018, his nadir PSA level was 1.368 ng/ml. Subsequently, it gradually increased again, and MRI showed that the size of the lesion in the right margin of the prostate increased. For histological confirmation, template needle biopsies of the prostate were performed in December 2019. Based on the pathological findings, 2 of 64 biopsy sites were cancer positive (Fig. 3). The patient was diagnosed with adenocarcinoma (Gleason score 4 + 4). At that time, CT scan, MRI, bone scan, and positron emission tomography revealed no metastatic lesions. Therefore, he was referred to our department for salvage surgery. He was diagnosed with localized prostate cancer after salvage HIRT. Thus, hormone therapy or sRARP was considered. He selected sRARP despite sufficient knowledge that the procedure is challenging to perform.

In April 2020, he underwent sRARP after two sessions of HIRT. The operative time and console time were 3 h, 31 min and 2 h, 53 min, respectively. The volume of blood loss was 100 ml.

**Figure 4:**
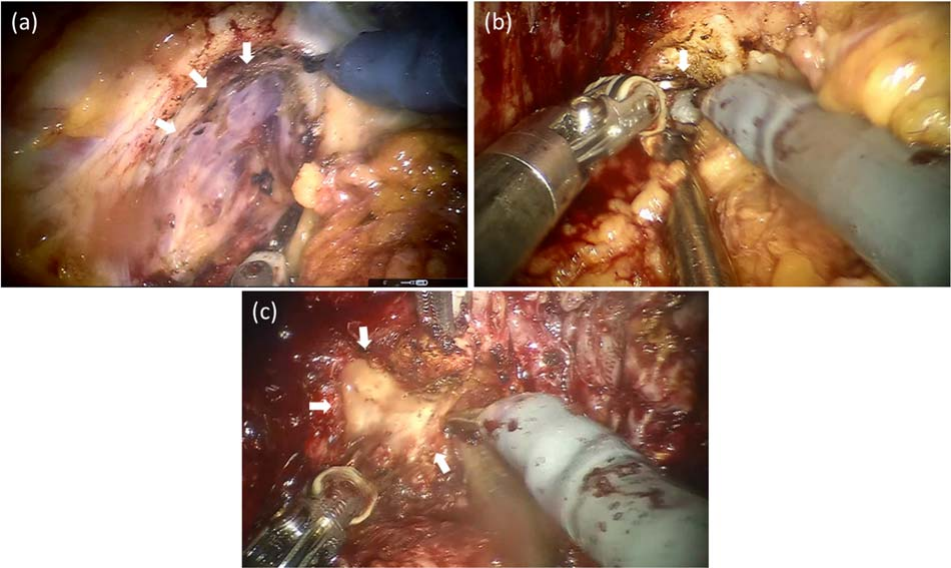
Operative findings after salvage robotic-assisted radical prostatectomy. The left endopelvic fascia had a strong adhesion to the levator ani and was challenging to divide (a). The retrotrigonal layer forming the left side had an extremely strong adhesion and could not be identified (b). The back part of the prostate had the strongest adhesions. The left ampulla of the vas deferens was almost dissolved and could not be identified (c).

The operation findings and technics were followed (Fig. 4). The surgery was performed using the intra-abdominal approach. After dividing the Retzius space and the fat tissue in front of the prostate, bilateral endopelvic fascia incisions were made. The right endopelvic fascia had moderate adhesions, and the left endopelvic fascia presented with strong adhesions (Fig. 4a). The vesico-prostatic junction was divided with the lateral approach. The retrotrigonal layer was easily identified from the right side. However, the left side had an extremely strong adhesion and could not be identified (Fig. 4b). Finally, bladder neck sparing was conducted from the right side without bladder neck reconstruction. Extremely strong adhesions around the bilateral ampulla of the vas deferens and the back of the prostate were observed, which were the most difficult points in this surgery. Nonetheless, the prostate was ultimately removed without major intraoperative issues (Fig. 4c).

Thereafter, Rocco, bladder urethral running, anterior, and bladder neck suspension suturing was performed with 3-0 Monocryl. A 16-Fr Foley catheter was introduced into the bladder, and a 7-mm silicone drain was placed in the pelvic cavity.

Urethro-cystography was performed 7 days after sRARP, which showed that there was no leakage. The urinary incontinence risk was low after removing the Foley catheter without acute urinary retention. He was discharged from the hospital on the 9th day after RARP.

**Figure 5:**
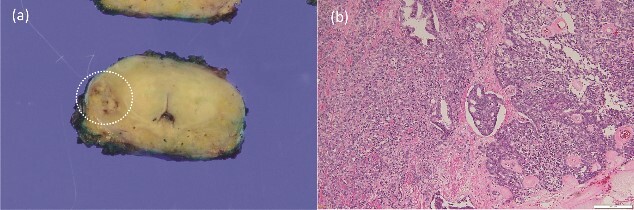
Macroscopically, infiltrating tumor cells with a maximum diameter of 13 mm were observed in the right peripheral zone (a). Histopathological examination showing invasive prostatic adenocarcinoma and hematoxylin and eosin staining at a magnification of ×100 showing perineural invasion and lymph node invasion (b).

The pathological finding after sRARP was adenocarcinoma (Gleason score 4 + 5, EPE1, RM0, ly1, v0, pn1, sv0, and pT3b). The lesion location (template biopsy-positive sites) matched well with the MRI findings (Fig. 5). The prostate capsule was fibrous, and it thickened owing to the HIRT.

His PSA level was undetectable 8 months after the surgery. Thereafter, his PSA level had gradually increased again. In March 2021, his PSA level increased to 0.104 ng/ml. Thus, goserelin acetate was introduced as adjuvant hormone therapy (Fig. 4). Then, his PSA level remained undetectable, and urinary incontinence and other complications were not observed 2 years after the surgery.

## Discussion

In approximately 50% of cases, recurrences after first-line nonsurgical treatment are localized to the prostate [2]. Salvage radical prostatectomy has better outcomes with the increased rate of robotic surgery. Paolo Gontolo *et al*. showed that robotic-assisted surgery had better outcomes in terms of volume of blood loss, length of hospital stay, and incidence of anastomotic urethral stricture rates [3].

The HIRT may have less anatomical effects on the periprostatic area because of a more precise irradiation than other nonsurgical therapies [4]. As in the case of conventional sRARP, adhesions are often stronger than the contralateral side, which is where cancer recurrence is found. In this case, recurrence occurred from the right lobe, and an extremely strong adhesion on the left side was observed. Hence, the left lobe could have been sufficiently irradiated.

sRARP generally requires more skill than regular RARP. However, HIRT may be more accurate and less periprostatically anatomical than other nonsurgical therapies.

Compared with normal RARP, sRARP is associated with prolonged postoperative hematuria and a higher incidence of anastomotic leakage, worsening of urinary continence, and other complications. Hence, the indications of sRARP after the HIRT should be considered. Nevertheless, it can still be a treatment option.

## Data Availability

Data cannot be shared for ethical/privacy reasons.
